# Foreign Medical and Physical Science and Literature

**Published:** 1819-02

**Authors:** 


### FOREIGN MEDICAL AND PHYSICAL SCIENCE AND LITERATURE.

THE character which the physiological and pathological doctrine of M. Broussais has assumed, and the degree in which it is estimated by many physicians of the first eminence in France, render a knowledge of it of general interest, and, indeed, of considerable importance. Since the publication of his *Examination of the Medical Doctrines generally adopted*—or, perhaps, rather, since his work *on Chronic Inflammations*—showed the depth of his views; his opinions have progressively obtained increased attention; particularly from those physicians who think that physiology is the only true foundation for medical knowledge.

It is somewhat remarkable that philosophers of the present day should, in attempting to refute a system of doctrine, direct their attacks against some few of its particular applications, and not against the principles on which it is founded: such, however, has been the conduct of the chief of the adversaries of the system of Dr. Broussais. They have thus either tacitly acknowledged the truth of its principles, or have felt their inability to produce substantial arguments against them. Some of the applications which the author has made of his theory are, doubtless, incorrect, or not clearly developed; but this should not be adduced as an objection to it on the whole; since both the adversaries and partisans of a system, the applications of which are so extensive, must perceive that the labours of a whole life would not be sufficient for its perfect illustration.

As this doctrine is, we believe, not generally known in England, we have considered that an exposition of it would be both gratifying and useful to our readers; and we had just began to arrange our observations, and to become sensible of the great length of time and labour that would be required to consider with sufficient attention the objections of its adversaries; and to digest the ideas of the author, and comprise his principal illustrations of them within concise limits, when a Memoir, embracing these objects, appeared in the second volume of the Journal complementaire du Dictionnaire des Sciences Medicales, executed with so much conciseness, precision, and judgment, that we could not hesitate to adopt the reflections therein adduced; since they enable us to lay the subject before our readers without further delay, and in a manner that we should despair to be able to excel, were we to proceed further in our own labours.

" The adversaries of the new doctrine, who are versed in the knowledge of the different and almost innumerable opinions that have successively reigned in medicine, may, perhaps, object to our designating under the term of the *Doctrine of M. Broussais* the series of opinions which that physician professes. We are not ignorant that several ideas are met with in authors more or less ancient, which have a great analogy with those of Dr. Broussais; we know that many notions by which he has profited are to be found in Baillou, Sydenham, Chirac, Bordeu, and Brown; and many of the physiological principles he teaches were advanced by Lorenz, Lamarck, and some other authors; and, therefore, we do not pretend that all his ideas are absolutely new: but we think, with him, that the greater number of those which are not so were misunderstood, or considered as incorrect, by the generality of modern physicians. We would also observe, to those who think it possible that a person can go from book to book to seek individually the ideas which he may adopt for his use, that they do not comprehend the manner in which knowledge can be acquired; they do not perceive the difference there is between detached propositions, often lost in a mass of absurdities, and a regular system of medicine: an entire life would not be sufficient for the researches alone that such a method would require. We may, therefore, attribute to the new system the name of M. Broussais; for, admitting that many parts of it previously existed, it is not less incontestible that it is he who has united them into a regularly-organized doctrine. In taking advantage of the labours of his predecessors, he has only imitated Hoffman, Stahl, and Böerhaave, whom no one has blamed for having acted in this manner. Much of the system of M. Broussais has been derived from Bichât, who himself profited so well by the beautiful ideas developed by Bordeu, in his Researches on the Glands and the Mucous Tissue. Bichât distinguished the particular structure and peculiar mode of vitality of each tissue of the animal economy. He considered that they might all be distinctly affected in their diseases, and that the phenomena which accompany their lesion are totally distinct in each species of tissue. He also perceived that no precise pathological knowledge could be acquired, until physicians made a knowledge of the diseased organ the basis of their study. But, notwithstanding the work of Bichât was in the hands of all practitioners, and the warmest eulogia were conferred on it, the ideas of that great man had remained sterile until they were adopted by M. Broussais.

The object of this physician is to apply physiology to pathology, and to refer to organic lesion all the diseases which result from functional derangement. He banishes from medical theory all ideas of primary general affections,—that is, those originally seated in all parts of the system. According to him, there is no agent which can directly produce disease of the whole system at the same time; the symptoms, more or less general, which have been considered as designative of such morbid affections, are only sympathetic effects of a primitive local organic derangement. In the course of our exposition of his opinions, we shall lay before the reader the facts which support this proposition. At present, it may be sufficient to observe, that, all parts of the body possessing different degrees of sensibility and excitability, and bearing a particular relation to a certain number of external bodies, it is not possible that all the organs can be simultaneously influenced by any one of them.

But to understand the mode of action of the system in the state of health is not sufficient to enable us to appreciate correctly the sympathetic influence which they exert on each other, and to arrive at a discovery of the diseased organ; "we must also," says M. Broussais, "determine why it is so,—in what manner it is so,—and how it may be possible to remove it: it is in that consists what we should understand by *the nature of a disease*;" and it is then alone we can proceed, with security, to a rational practice of medicine.

The human body is not an homogeneous mass; it is composed of numerous organs, united to each other by the three organic systems that enter into their composition, and which establish between them more or less intimate relations. These systems are the sanguineous, lymphatic, and nervous. In the first, it should be observed, that its most finely-developed extremities—that is, the capillary vessels—do not the whole of them give passage to red blood; a great part of these capillary vessels admit only colourless fluids, which serve to form new productions. From morbid phenomena presenting considerable differences according to the nature of the capillaries in which they are seated, M. Broussais designates the whole of the vessels of the body by the term *vascular system*, and distinguishes them into red and white vessels; the latter comprising the radicular lymphatic filaments, and the secretory, excretory, and other vessels, which never give passage to red blood.

The final divisions of the vascular and nervous systems are intimately connected with each other, on being distributed to and finally lost in the various organs; and the different proportions in which they exist give to these organs particular properties. Thus the red vessels and nerves are but sparingly distributed in the serous membranes, cartilages, bones, &c.; but they are more numerous in the mucous membranes, parenchymatous structure, and muscles; and in direct proportion to their predominance is the liability of the parts to inflammatory and nervous diseases. We must observe with M. Broussais, that the intimate connexion between the ramifications of the vessels and nerves is such that, in the great majority of cases, morbific causes extend their action from one to the other. Thus, although the nervous system receive the first impression, the irritation is almost always, sooner or later, conveyed to the capillary blood-vessels; the result of which may be inflammation, greater or less in degree. M. Broussais shows that, in a great number of affections regarded as essentially nervous, the increased sensibility of the nerves is, more frequently than is commonly supposed, kept up by vascular irritation; and that it is this we must attack in order to remove the disease. These important facts will, however, be considered, as well as the modifications that irritation undergoes when it is prolonged in an organ, when we examine those parts of the new doctrine which relate to inflammation, nervous affections, and organic lesions.

The vascular and nervous systems, we have observed, establish between the different organs such communications, that the impressions received by any one of them is rapidly communicated to the rest. The various phenomena that result from this mutual consent constitute sympathies, the study of which is of so much pathological importance, particularly in the doctrine of M. Broussais; and it is shown by this physician, that the nervous system is its exclusive agent. Indeed, however obscure this subject may have appeared to the generality of modern physiologists, it is evident that the different organs being connected only by vessels and nerves, and the former only supplying them with the materials for nutrition, it must be the latter which are the conductors of the impressions they transmit to each other. But, on admitting this proposition, the following question arises:—Is it necessary that these impressions should pass the cerebral centre? M. Broussais decides in the affirmative. According to him, the impressions received by the nerves, whether of the external parts of the body, of the mucous membranes, or the internal structure of organs, is always transmitted to the brain; whence it is reflected throughout the whole extent of the sensitive system. The effects that result from this rapid communication of impressions vary according to individual predispositions: if an organ be inflamed, it will always experience this influence in a particular manner; in other cases, the most sensible parts will be especially affected. Thus, in one person the stomach, in another the lungs, in a third the pleura, will be the principal seat of sympathetic disease from the influence of irritation.

It must be considered that the communications established between the principal viscera by the numerous branches of the great sympathetic nerve, with those which exist between the central organs and all parts of the body by means of the filaments of this nerve, which accompany the arteries until they are lost in the tissues to which they are distributed; serve to transmit directly the impressions from one to the other of these parts, at the same instant that they are imparted to the brain; so that the whole system may be simultaneously affected, and not *after* the cerebral centre. Thus, an irritated organ becomes (if it may be so expressed) a focus whence impressions more or less powerful diverge, that affect at the same time all the animal economy, and which concentrate their influence on the brain, or on other viscera, according as the sensibility of them may predominate. We must observe, that we do not affirm any thing on this point, but it appears to deserve attentive examination.

After having taken a rapid view of the whole animal economy, and considered in a general manner the relations that exist between its different parts, let us, with M. Broussais, devote our attention to the mode of action of morbific causes.

"Our body is maintained," he observes, "by appropriating to itself the substances which it has the power to assimilate to it; and in rejecting those which are either injurious, superfluous, or have been already employed. In order to execute these actions, it is endued with sensibility; by means of which all that comes in contact with it produces motions in its organization: there is also a necessity that it should be constantly excited; if it cease to be thus affected, it languishes, and at length perishes." The agents of this irritation are external bodies; the fluids contained in the vessels; and the influence which the organs reciprocally exert on each other. These different agents, M. Broussais remarks, may be distinguished into those which contain nutritive materials, and those which excite the parts without affording any thing which is capable of augmenting their substance. The inferences that may be deduced from this division will be hereafter developed.

In the state of health, each organ enjoys the sum of the powers which is necessary for its action; and, receiving from the rest of the system (M. Broussais comprises under this denomination the whole of the exciting and debilitating agents) a determinate degree of stimulating influence, it reverts, in like manner, an excitant power over the whole animal economy, which bears an appropriate relation to its wants. But this equilibrium may be destroyed, and that because the influence exerted on any particular organ may excite it either too little or too much. This constitutes a state of disease.

It is evident that irritation* will never be confined to the part which immediately experiences it, unless it be very feeble; but the difference in its degrees will produce different results from its transmission throughout the rest of the body. If the stimulus, although weak, be continued in an important organ for a long period of time, it then influences others, producing in them increased action. This is observed in the first stages of pulmonary irritation, in persons disposed to phthisis; in those who begin to indulge in too frequent exercise of the genital organs; in irritations of the stomach, &c. &c. In all these cases an extraordinary degree of activity may be observed in all the functions,—an excess of apparent health, which the attentive physician perceives to be only the prelude to the most serious diseases. But, when irritation is more considerable, and particularly when it is seated in the parenchymatous structure of an organ, or on a mucous membrane, (parts abundantly supplied with blood-vessels,) it produces an increase of action in all the other parts of the system; the result of which is termed *inflammatory fever*. We shall particularly treat of the mode in which fever is thus produced, when we consider the ideas of M. Broussais on this subject, on another occasion.

When local irritation is violent in degree, other phenomena are manifested in the system: the diseased part appears to draw to

---

* By irritation, M. Broussais would signify an exaltation of the action of the vessels or nerves which enter into the composition of the organs of the animal economy.

itself all the vital powers, leaving the other organs in a state of langour and weakness, which has been designated by the term *adynamia.* The facility with which this concentration of action may be produced, depends both on the nature of the part affected and the habit of body of the patient. Thus, the viscera contained in the splanchnic cavities, particularly the stomach and intestines, give rise to them much more readily than any other parts. Besides which, there are some persons in whom but trifling local affections will produce extreme prostration of strength; whilst others experience considerable re-action from more violent degrees of irritation. These distinctions, founded on physiological observation and the results of the practice of medicine, are, as we shall hereafter show, one of the bases of the doctrine of M. Broussais respecting the species of fever termed *adynamic.*

A question here presents itself of the highest importance in pathology:—Are morbid affections dependent on irritation of parts, more numerous and more frequent than those dependent on debility as their primary cause? M. Broussais decides in the affirmative. The study of diseases seems to furnish the most convincing evidence of the correctness of this opinion: "it proves that it is by inflammation destroying, with greater or less promptitude, one or more organs essential to life, that the greater number of persons perish."—"Every practitioner, accustomed to contemplate the ruins of the admirable edifice constituting man, which the efforts of the medical art have not been able to avert, must be fully convinced of this truth. On perusing the immortal work of Morgagni, we every instant perceive the most unequivocal effects of inflammation. If we question patients suffering from chronic disease, the greater number of them will be found to complain of fixed and permanent pain of some part; whilst the fever, and the state of general depression and decay, which we witness in them, lead us to anticipate what is but too often realized,—that they will perish from inflammatory disorganization of some one of the viscera."—"If we examine acute diseases, (continues M. Broussais,) we shall almost always observe symptoms of re-action, or of concentration of the vital powers, to coincide with inflammation of an organ, the lesion of which, though often visible during life, may not be discovered until after death."

*(To be continued.)*

---

### *A Memoir on Temulent Diseases;* by Joseph Klapp, M.D. Physician to the Philadelphia Infirmary.

It has lately been remarked by Dr. Edward Percival, that "it is peculiarly necessary to assert the value of physical researches at the present day, when certain notions respecting the paramount efficacy of *moral management* of the insane are growing into prevalence. No doubt, indeed, can be entertained of the importance of this auxiliary, both in a humane and remedial aspect; yet, if the labours of pathology be sacrificed to the light suggestions of disappointment or scepticism, the certain effect will be the retrogradation of science." The same disappointments in pathological enquiries have influenced the reasonings of physiologists on the nature, as well as the treatment, of mental alienation; and attempts have been made to explain the phenomena of that derangement by arguments deduced solely from metaphysical principles. But, does not the description of De Bonald point out the utmost degree to which our ideas should be suffered to stray from materialism in our enquiries into the animal economy, when he happily describes man as "an intelligence *served by organs?*" We have not, however, yet arrived at that period when imaginary agents, to which powers and functions *ad libitum* are attributed, will cease to be called in to our aid, in order to get rid of every difficulty that may arise in the course of our enquiries. Thus, a professor, who is acknowledged throughout Europe as a man of considerable learning and talents, has lately described delirium as "a state in which reason is *eclipsed* by some derangement, *direct or indirect*, of the *intermediate substance* which is subservient to the relation existing between intelligence and the bodily organs."*

We consider the Memoir of Dr. Klapp, which we are about to lay before our readers, of considerable value; not only from its furnishing testimony in support of what we have just endeavoured to show the importance, but also because it adduces evidence of another point that has been too much neglected—the *gastric origin* of mental insanity, and several other of the diseases usually termed *neuroses.*

Physicians have, indeed, for some time past been disposed to seek for the origin of several *nervous* maladies in disorder of the chylopoïetic viscera; but those views have not, in general, been sufficiently extended to mental alienation, notwithstanding the facts which have, during some years past, been adduced in support of them by several physiologists of great research and accurate observation. The writings of Housset, Pinel, Prost, Esquirol, Kaempff, Schmucker, Hufeland, Elvert, Cheyne, Hamilton, Cox, Percival, and many other physicians who have had opportunities for extensive examination of bodies after death, furnish sufficient evidence to prove that mental disorders most frequently arise from the source to which we have alluded. Pinel, whose observations and opinions merit the greatest attention and respect, expressly states that "the primary seat of disease productive of mental alienation appears to be, in general, in the region of the stomach and intestines, whence it is extended, as from a centre, by irradiation, and deranges the intellectual

---

* "Le delire est un état dans lequel la raison est éclipsée par un dérangement quelconque, direct ou indirect, de la substance intermédiaire qui sert aux relations entre l'intelligence et les organes corporels."

functions."* Notwithstanding the testimonials to which we have referred, the brain is still too much regarded as the original seat of the disease on which those maladies depend; a disposition that appears to have been much increased by the opinions of GALL and SPURZHEIM; and which may probably be placed in opposition to the numerous advantages that physiology has derived from their doctrines.

Dr. Klapp commences with observing, that—

"Some late particular experience induces me to solicit the attention of the faculty to the pathology, but more especially to the treatment, of those kinds of vertigo, mania and epilepsy, which are produced by the inordinate use of ardent liquors. They are, I believe, equally gastric in their origin, and so similar in nature as will justify a declaration of their being only different members of the same group or family of diseases. That these three complaints are derived from the same or a similar morbid state of the stomach, induced by its having been unduly stimulated with ardent liquors, is a position which I trust will prove to be well founded.—1st, On the sameness of their remote cause; 2d, on their symptoms; 3d, on certain facts; 4th, on dissections; and 5th, on their method of cure.

"In vertigo, the dyspeptic symptoms more constantly attend. Epileptic patients are, I believe, always more or less harassed with nausea. In the cases which will be presently related, frequent reachings, or ineffectual vomiting, constituted a prominent and leading symptom. In the first and convalescent stages of drunken insanity, it is well known that patients refer their complaints chiefly to the stomach.

"Certain facts have come within my knowledge, which I think will be admitted to afford some support to our gastric pathology. These have a tendency to show that, in habits predisposed to the epilepsy and mania under consideration, irritants of different kinds are capable of exciting those diseases. The doctrine also derives additional support from the circumstance of those diseases almost immediately ceasing to exist upon extracting from the stomach their noxious causes. The first of the facts alluded to I will transcribe from a case of mania a temulentia described in my communication on that disease in the seventh volume of the Eclectic Repertory.—'20th of August, while the patient was convalescent, he requested to have something to eat: I would only permit him to have panado or gruel containing a small quantity of brandy, and he was allowed for drink some weak wine-and-water.—21st. My directions had been deviated from; the patient was indiscreetly permitted to eat several small birds; shortly afterwards he began to complain of a sense of heaviness, and of pain in the region of the stomach and back. In the night, slight but evident aberrations of the mind were again noticed, and his rest had not been so

* *Traité Medico-Philosophique sur l'Alienation Mentale*, p. 141.

comfortable. Suspecting that the stimulus of his yesterday's dinner had occasioned these symptoms, I desired that the emetic solution should be again given to him.

"22d. The medicine had operated freely after giving the first dose: the uneasy feelings about the stomach from that time ceased, and he slept comfortably through the night.

"23d. The patient to-day was found walking about the house in every respect well, and the attendance was discontinued.'

"This case exhibits, I think, very clearly a specimen of derangement of mind, symptomatic of a primary disease of the stomach, induced by the irritating effects of aliment which it was incapable of digesting. As soon as the emetic had relieved the pain and oppression about the stomach, by the displacement of their cause, the mental disease disappeared.

"The second fact I will submit, of an import, and admitting of a construction, similar to the first, I have been supplied with in the case of a patient who had been cured of lunacy from intemperance in the infirmary of the alms-house. While convalescent he was liberated from his cell, and permitted to walk in the yard. Shortly after his enlargement he privately ate a quantity of hard, indigestible bread, and then drank freely of cold water. He soon began to complain of his meal sitting heavy on his stomach; derangement of mind speedily supervened, and it became necessary to remand him to his cell. The cause of his disease having been satisfactorily discovered, an emetic was administered, which operated briskly, and brought off the bread he had eaten; and shortly afterwards he became again rational.

"A third patient, with the same disease, while convalescent, was induced, through the persuasion of some anxious officious friend, to take a raw egg with wine in the morning to strengthen the stomach: the dose proved indigestible; his stomach soon became uneasy, and epileptic convulsions, with derangement of mind, ensued. In the course of a few hours, after many remedies had been ineffectually applied, he took something (at present not recollected what) which acted as an emetic, and the egg was disgorged entire, or nearly as much so as when it was swallowed. After this the epilepsy and mania ceased, nor had he any return of them from that time.

"If it be admitted that the diseases, whose pathology I have been endeavouring to illustrate, consist of a morbid condition of the stomach, the indication of cure from thence to be deduced is rendered very evident: to remove offensive matters which impress a morbid influence on the nerves of that organ, and more especially to alter its state so as to enable it to perform its healthy functions, the use of emetics is clearly indicated. The subjoined cases will exhibit the effects of this practice. I will begin with vertigo.

"Mr. ——, about 50 years of age, has long been in the habit of using strong drink to an excess. On the 1st of June last, my assistance in his case was desired, and of which he gave me the following account. For a length of time he had been subject to bilious pukings in the mornings, a poor appetite, and vertigo of a severe kind. The last affection often seizes him while walking about, and sometimes induces a degree of debility in his limbs, nearly causing him to fall. This morning he suffered a severe attack of this kind, in consequence of which he fell on the floor; though the disease did not extend so far as to occasion either convulsion or a loss of sense at the time. His skin was cool, and the pulse nearly natural, beating about 63 times a minute.

"He was directed to take two grains of tartris antimonii, dissolved in a little warm water, every fifteen minutes, until the stomach was freely evacuated, and, while operating, to drink warm water copiously. June 2d, on entering his chamber, he accosted me with the following remark:—'I am much better to-day, and have a strong appetite, which I have not before known for a long time.' Eight grains of the tartar emetic had been taken when vomiting ensued, and the stomach was well deterged of its morbid contents; it also purged him twice freely. When the operation was over, he found his vertigo was gone. The pulse this day stood at 84, and somewhat weak; his skin remains cool, and he now complains of nothing but a sense of general weakness. He was ordered a light diet, and the use of bitters.

"June 3d. Has had no return of the vertigo, and his appetite is unimpaired. Continue bitters.

"June 4th. Remaining well, further attendance was omitted."

Dr. Klapp then refers to the seventh volume of the Eclectic Repertory for several cases, related by him, of mania à temulentia, which were successfully treated in the same manner; one of which we shall transcribe:—

"On the evening of the 2d of October, 1814, G—— was admitted a patient into the alms-house, for mental derangement of the furibund grade, and satisfactorily ascertained to have been occasioned by the excessive use of ardent liquors. He is a carpenter by occupation, is about 40 years of age, and on enquiry I was told that for many years he had been a very intemperate man. To prevent injury to himself, it was found necessary, in addition to the use of the strait-waistcoat, to have him closely chained to the floor of his cell. His pulse was frequent and weak, and the trembling of his limbs was very great. He took two grains of the tartris antimonii, dissolved in a little warm water, every fifteen minutes; vomiting was not created until after twenty grains had been administered, when a large quantity of viscid mucus and sordes were brought up, and afterwards the bowels were smartly purged. After the operation of the antimony was over, the pulse had become less frequent, and fuller. The patient had some sleep during the latter part of the night.

"3d. He seems much better; pulse remains as last stated; he speaks correctly on some subjects, but remains evidently deranged on some others. On entering his cell, we found him very curiously employed; he was pressing with both arms, and apparently with all his strength, against the wall. I requested an explanation of his conduct, but he refused to give me his reason for doing so. The cell-keeper afterwards informed me, that during the whole morning he had been bearing against the wall, through a fear (as G—— had stated to him) that, unless it was held up, it would certainly fall upon him and crush him to pieces. I desired the tartarised antimony to be repeated.

"4th. When sixteen grains had been given, first vomiting, and then copious purging, ensued. On examination, the pulse proved to be 100, and weak: he is plainly more rational in both conversation and behaviour; in short, it was rather difficult to discover that there was any of the mental affection remaining. In the course, however, of my conversation with him, it was perceived that his intellectual faculties had not yet been perfectly reinstated. Prescribed blister-plasters to the ankles.

"5th. Out of the last twenty-four hours he has slept eighteen, and is still inclined to sleep. When questioned about sleeping so much, seems conscious of his indolence, and apologised for it by saying that the narrow limits of his cell would not admit him to take exercise, so he thought he might as well sleep as not. The blisters had not yet been dressed. By the test of conversation, he this day proved rational upon every subject. The tremors of the limbs have totally ceased; pulse remains rather too frequent, but has more strength than at any time heretofore. Nothing prescribed but some light diet.

"6th. As yesterday. On entering the cell, I found G—— was still very comfortably reposing in bed: he stated to me that his sleep had been uninterrupted through the night; and asserted that, with the exception of a little weakness, he now felt as well as he had ever done, and requested his discharge; but was prevailed on to delay his departure a day or two longer. The pupil of the house was directed to furnish him with a little spirit and water for drink, and to give him panado, containing some spirits, for sustenance.

"7th. Continuing to be well, I consented to his liberation."

In a Memoir published in the work to which we have referred, Dr. Klapp expressed an opinion that the use of emetics in mania à temulentia accompanied with epilepsy would be unsafe; he has since, he observes, become convinced that this was entertained without foundation. From extensive experience, since the date of that publication, he has ascertained that, so far from the accompaniment of epilepsy forming an objection to the use of the emetic, that disease, when the effect of hard drinking, whether it occurs in conjunction with mania, or exists in a separate state, is itself safely and promptly removed by it. Several cases are adduced to illustrate this remark, of which our limits will only permit us to transcribe the following:—

"T. F. aged 47 years, for a length of time in the habit of drinking to an excess, during the last week or ten days has been affected with sleepiness, a loss of appetite, pain in the breast, and tremor in the limbs.

"On the 21st of May of the present year, at ten o'clock in the morning, he was suddenly seized with a violent epileptic fit, which continued fifteen minutes.

"On the 22d, he fell into a state of mental derangement; and,

"On the 23d, I found him labouring under several diseased perceptions, particularly from sight and hearing. His false perceptions and insane thoughts are of this kind:—He is constantly engaged in picking up, in different parts of the room, certain imaginary objects, and seems desirous of having them all together in one place. He charges his wife with having conspired with a number of persons for the purpose of killing him, and to carry off his property. He frequently declares that, during the whole of last night, his house, in consequence of the efforts of these conspirators to plunder him of his property and to carry him off, was made a constant scene of bustle and contention. His skin is covered with a cool, or rather a cold, perspiration; the tongue is partially covered with a brown fur; the pupils of his eyes are a little dilated; has no pain in the head. Through a fear that I was actuated by some improper motive towards him, he would not permit me to have his hand long enough to feel his pulse. I ordered two grains of tartarised antimony to be given to him, dissolved in water, every fifteen minutes, till vomiting was freely excited; after which, to have him kept as quiet as possible to encourage sleep.

"24th. After three doses of the antimony had been given he vomited considerably, and discharged a large quantity of a dark-green coloured sordes. When the operation was over, of his own accord he went up into his chamber, laid down, and slept a little. When he awoke, his insanity continued, but to a less degree than before. He was very restless through the night; and this morning, before I saw him, his wife again gave him the emetic, which operated both upwards and downwards severely. Neither yesterday nor to-day, while the emetic was operating, could any disposition be perceived to a return of his epilepsy. Mrs. D. this day assures me that, each time after using the emetic, she could perceive an evident abatement of his mental derangement. For the first time, to-day he would permit me to examine his pulse: it was 134 a-minute, and possessed some force. His skin remains moist, and has acquired its natural temperature; the tongue has become clean and moist. A blister-plaster was directed to be applied to the back of his neck. A light diet was enjoined, and the use of spirituous drink forbidden.

"25th. When I entered his room to-day, he said a great change had occurred in his case since yesterday; a remark with which I was pleased, as it indicated a return of reason. I asked him if he recollected what was the matter with him yesterday? his reply was, that he believed he had been out of his senses. For a short time to-day he was again deranged, but afterwards he went into a sound sleep. When he awoke he was sensible, and has since remained so. The pulse is now only 100, and the tongue and skin natural. A continuation of rest and the light diet were directed.

"26th. In mind he now appears to be quite well, but complains much of weakness and a soreness in all his limbs. I prescribed half a cupfull of bitter tea to be taken several times through the day. The tea was made by infusing bark, serpentaria, and chamomile, of each two drachms, in a quart of boiling water.

"27th. To all appearance being at this visit well in both body and mind, I concluded to take my leave of Mr. D."

---

*Cases illustrative of the beneficial Influence of Nux Vomica in Paralysis.* Related by Dr. Lescure.

A young man, who had been afflicted with epilepsy from the age of twenty, which was first induced by the influence of terror, was twelve years afterwards attacked with *paraplegia* from a similar emotion: this new affection entirely superseded the former. Four years passed away without his experiencing any relief from the measures that were employed, when he came under the care of Dr. Lescure. Four grains of the extract of nux vomica were administered daily, divided into two doses. After eight days the dose of the medicine had been raised to five grains, and to six grains in the course of the following week. The patient then began to complain of heat about the stomach; a sense of constriction in the abdomen; difficulty in passing the urine; and occasionally a sense of contraction in the muscles of the lower extremities. The use of the remedy was suspended for a short time, and then resumed in the former quantity. Three weeks afterwards the retention of urine returned, and was more complete than on the former occasion; and it was accompanied with forcible sudden contractions in the paralyzed limbs. These symptoms disappeared on omitting to use the extract for four days: at this time the patient was able to leave his bed, and walk about his chamber with the assistance of an arm. The medicine was then repeated in doses of eight grains: at the end of three weeks he experienced an attack of tetanus, which continued for four hours. This was followed by a still greater amelioration of the original disease, and at length by its perfect and permanent removal.

A female, who had been subject to attacks of hysteria and pains about the loins and thighs, was seized with paraplegia after one of those paroxysms. The extract of nux vomica, conjoined with opium, was directed for her by Dr. Lescure. During the first week, the quantity administered daily of the former had been carried to six grains, that of the latter to half a grain: one grain and the sixth of a grain, respectively, were each week added to the daily doses of the medicine. At the end of a month the paralyzed limbs became affected with slight contractions, to which succeeded a return of sensibility. On the forty-third day, pain about the stomach, and difficulty in swallowing and passing the urine came on; which accidents were followed by swelling of the paralytic extremities. These symptoms continued to increase for three days, when the use of the remedy was suspended. The same mode of treatment was resumed on the disappearance of these symptoms; but fifteen days afterwards they returned. On their subsidence the medicine was again administered; and eight days afterwards the patient experienced convulsive motions of the limbs, followed by tetanus and retention of urine. An amelioration of the original disease was regularly noticed after each of these attacks, and after the last the patient began to walk. The paralysis then gradually disappeared, and her health became perfectly re-established.

A female, who had become affected with paralysis of the whole of the limbs, incontinence of urine, and amaurosis, subsequently to the cessation of the menses, and which continued to become more fully established, took the extract of nux vomica for two months. During this time two of the attacks above described, and which appear attributable to the medicine, had taken place. At the end of that period the disorder had entirely disappeared.

The following case of paralysis, treated by the same remedy, is related by Dr. Finot, of Luxeuil:—

L. A. aged 52 years, of a robust habit of body, who had led an indolent life and indulged in excess in the use of spirituous liquors, was seized, in the month of February, 1818, with an attack of apoplexy, which was followed by hemiplegia of the left side. All the usual measures were employed, without being productive of benefit. On the 1st of June he consulted Dr. Finot. He then had hardly any power of motion of either the arm or leg of the affected side; his speech was hardly intelligible, and the motion of one side of the tongue appeared to be performed with much difficulty. There was a distortion of the mouth, and complete eversion of the inferior eyelid of the left side.

On the 2d of June he took four grains of the powder of nux vomica; the quantity of which was increased without being productive of ill effect until the 8th, when slight spasms and convulsions of the affected limbs took place; which were succeeded by heat and pain of the parts, and greater facility of motion. The quantity of the remedy daily administered had been carried to twelve grains on the 14th of the same month, when the patient suffered darting pains and tremblings in the affected side. On the 18th, he could use the arm tolerably well, and bend the leg on the thigh, and the latter on the pelvis. On the 22d, he had recovered the power of almost the natural motion of the arm and leg, and could pronounce words with but little difficulty. The distortion of the mouth was hardly perceptible. On the 27th, after very painful darting sensations, which recurred during several successive days, the eyelid recovered its natural position. On the 30th, the patient walked with the assistance of a stick, experiencing only a little stiffness of the limbs. On the 4th of July he was perfectly well. The dose of the powder of nux vomica had been extended to twenty-four grains during the last few days.*

---

*Arthrocacology; or a Treatise on Luxations of the Joints dependent on internal Causes; and on the Efficacy, Mode of Action, and Application, of the Actual Cautery, in the Treatment of those Diseases.* By JOHN NEPOMUK RUST, Doctor in Medicine and Surgery; Professor of Medicine and Surgery in the Royal Military Academy at Berlin; Director of the Hospital of Charity, &c. &c. 4to. pp. 195. Vienna, 1817.†

"*Ex ruinis alienæ existimationis sibi gradum ad famam struunt,*" as Scaliger observes, is too often applicable to the followers of science: and this not so much from an ambitious impulse, as from the charms of novelty of observation and opinion giving to certain objects such a degree of interest and importance, as to lead them to undervalue every thing that may not appear subservient to those objects and tend to favour their attainment. The chief intention of Professor Rust, in the treatise which we are about to introduce to our readers, is to show that all the notions previously held of the causes of spontaneous luxation of the joints have been erroneous, and to advance the opinions respecting it which his own observations and experience have led him to adopt. We may here mention, that it is not our intention to enter into a general analysis of this work; we shall merely adduce an abstract of those parts which are of importance, either from the novelty of opinion, or the pathological facts to which they relate: but, in justice to the author, we must remark, that the results of very extensive pathological and medical observation which it details, and the depth of erudition which it evinces, will alone render it an highly valuable addition to medical literature.

After noticing, in succession, the opinions respecting the causes of what is usually termed "the hip-joint disease," which have been entertained by former writers; and showing that it has been attributed by Schwenke, Gorter, Vermandois, Portal, Callisen, Plenk, Tschep, Monro, and De Haen, to inflammation of the synovial glands; by Clossius, to that of the articular capsule; by Duverney, Albers, Dorner, Russel, Boyer, and Ficker, to inflammation of the soft parts, and ulceration of the cartilage; by Camper, Van Swieten, and Freke, to alteration in the state of the synovial fluid; by Van der Haar, Portal, Kortum, Hagedorn,

---

* *Journal Universel des Sciences Medicales*, tome xi.

† *Arthrokakologie; oder über die Verrenkungen durch innere Bedingungen; und über die Heilkraft, Wirkungs—und Anwendungsart des Glüheisens bey diesen Krankheitsformen.* Von Johann Nepomuk Rust.

Heister, Platner, De Haen, and Richter, to a disease of the articulating surfaces from metastasis of an affection of some other parts; he endeavours to prove, from clinical observation, analogy, and examinations after death, that this malady commences by an inflammation and tendency to ulceration of the internal medullary membrane (*periosteum internum,—tela medullaris* of Blumenbach,) of the ends of the long bones; which, extending into their substance, produce enlargement, and subsequently caries, of their articulating extremities; and that it is from this enlargement rendering them disproportionate in bulk to the articulating cavity, that dislocation takes place.

This description Professor Rust would apply to all the affections which are usually designated by "the hip-joint disease;" but we are inclined to suppose that it relates solely to what is generally considered as scrofulous inflammation of the bones, and that a distinction should be made between this disease and that which is described by Mr. Brodie, and some former writers, as commencing in the cartilages of their articulating surfaces. That an affection of the latter kind does occasionally happen, is shown in the abstract we have given from the work of Mr. Brodie; but there may exist a doubt, as that gentleman has expressed, whether such is the ordinary origin of the disease. Yet it appears somewhat extraordinary, that Professor Rust should not have met with a single instance of this kind in his extensive experience. This may, perhaps, have arisen from the few opportunities that occur for immediate examination of the parts in the early stage of the malady, when such a distinction could alone be made; and those few opportunities which he has been able to embrace may, probably, have led to error, in consequence of their happening in patients who have died from diseases, where that under consideration has only been one of several symptoms, which were in reality of a scrofulous nature. Thus, in one instance of this kind related by Professor Rust, the seat of the malady was that which we have described: this was in a girl who died at twelve years of age from scrofulous pulmonary consumption, (an einer skrophulosen lungen-schwindsucht gestorben war.) We shall detail more fully the observations of the author on this part of the subject, in order that our readers may be furnished with grounds for the formation of their own opinions respecting them: but we discover that the near approach already made to the final limits of the present Number of our work obliges us to postpone this undertaking to a future occasion.

